# Synthesis of Thieno[2,3-*b*]thiophene Containing *Bis*-Heterocycles-Novel Pharmacophores

**DOI:** 10.3390/ijms14035712

**Published:** 2013-03-12

**Authors:** Yahia Nasser Mabkhot, Assem Barakat, Abdullah Mohammed Al-Majid, Muhammad Iqbal Choudhary

**Affiliations:** 1 Department of Chemistry, Faculty of Science, King Saud University, P. O. Box 2455, Riyadh 11451, Saudi Arabia; E-Mails: amajid@ksu.edu.sa (A.M.A.-M.); iqbal.choudhary@iccs.edu (M.I.C.); 2 Department of Chemistry, Faculty of Science, Alexandria University, P. O. Box 426, Ibrahimia, Alexandria 21321, Egypt; 3 H.E.J. Research Institute of Chemistry, International Center for Chemical Sciences, University of Karachi, Karachi 75270, Pakistan

**Keywords:** thienothiophene, DPPH radical scavenging activity, β-glucuronidase inhibition, α-glucosidase inhibition, cytotoxicity, cancer cell lines

## Abstract

Thioenethiophene derivatives represent an important class of compounds with diverse biological activities. We describe here the synthesis of a new series of thieno[2,3-*b*]thiophene containing *bis*-heterocyclic compounds **3**–**7**. All the compounds were evaluated for their *in vitro* antioxidant potential, α-glucosidase and β-glucuronidase inhibiton and anticancer activity against PC-3 cell lines. Compounds **2b** (IC_50_ = 1.3 ± 0.2 μM), **5a** (IC_50_ = 2.3 ± 0.4 μM) and **5b** (IC_50_ = 8.7 ± 0.1 μM) showed a potent inhibition of β-glucuronidase enzyme, more active than the standard d-saccharic acid 1,4-lactone (IC_50_ = 45.8 ± 2.5 μM). Compounds **5a** (IC_50_ = 22.0 ± 0.3 μM) and **5b** (IC_50_ = 58.4 ± 1.2 μM) were also found to be potent α-glucosidase inhibitors as compared to standard drug (acarbose, IC_50_ = 841 ± 1.7 μM).

## 1. Introduction

Thienothiophene derivatives possess diverse biological activities, such as antitumor, antiviral [1–7], and antibiotic properties. They are also used as antiglaucoma drugs and as inhibitors of platelet aggregation [8–12]. Recently, numerous studies have been conducted on biological profiles of thieno[2,3-*b*]thiophene scaffold. Chemically these compounds have also attracted considerable attention as moieties that offer significant synthetic advantages, such as centrosymmetry and higher rigidity. They are also used in the design of novel nonlinear optical (NLO) systems through incorporation within unsymmetrically functionalized cyclophane [2]. Thienothiophene derivatives have been vigorously investigated from various viewpoints, *i.e.*, unusual physical, chemical, biological properties [13–16]. On the other hand, imidazopyrimidine and triazolopyrimidine analogs can be suitably modified with the introduction of different heterocyclic moieties to confer a broad range of biological activities, such as antimicrobial effects [17–21].

**Scheme 1:**
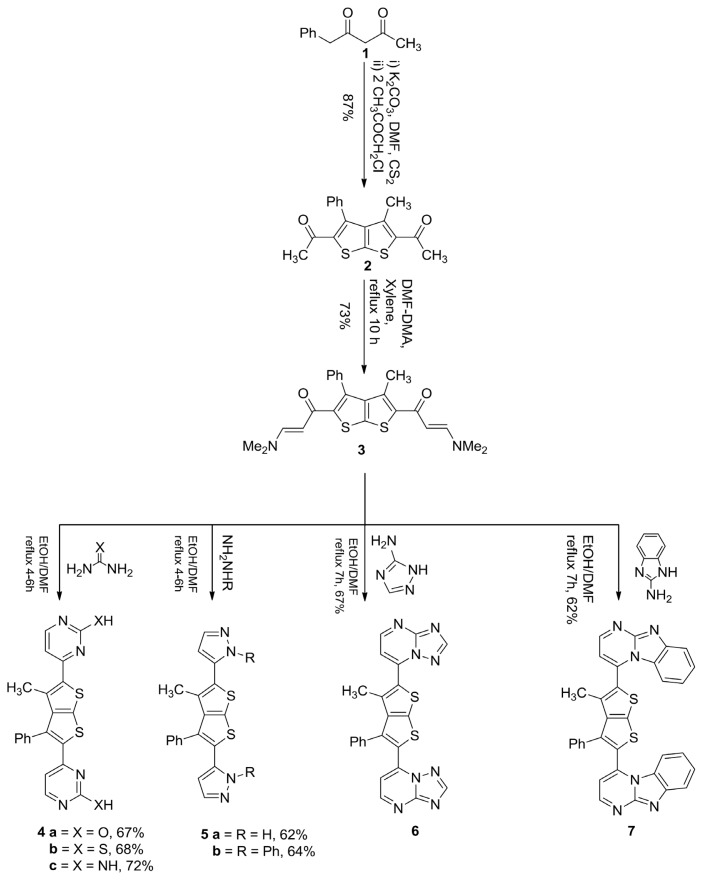
Synthesis of compounds **2**–**7**.

As part of our continuing efforts toward the synthesis of bisheterocycle system and convenient access to the diverse frameworks present in them, we have explored the synthetic potential and generality of a cyclization strategy to gain facile entry into the thieno[2,3-*b*]thiophene framework from a conveniently accessible precursor as shown in Scheme 1. Our new results on the thieno[2,3-*b*]thiophene derivatives, including chemical synthesis, their *in vitro* antioxidant potential, α-glucosidase and β-glucuronidase inhibition, and anticancer activity against PC-3 cell lines are presented herein.

## 2. Results and Discussion

### 2.1. Chemistry

The synthesis of the pyrimidine, pyrazole, triazolopyrimidine and imidazopyrimidine derivatives **4**–**7** were carried out by reacting commercially available benzoylacetone **1**, dimethylformamide dimethylacetal (DMF-DMA), 4-amino-1,2,4-triazole and 2-aminobenzimidazole. Previously 1,1′-(3-methyl-4-phenylthieno[2,3-*b*]thiophene-2,5-diyl)diethanone **2** was synthesized starting from benzoylacetone **1**. The method consists of sequential directed nucleophilic addition, side chain deprotonation, nucleophilic addition, and cyclization using a nitrogen or sulfur moiety as internal nucleophile. Condensation of **2** with dimethylformamide dimethyl acetal (DMF-DMA) under reflux for ten hours in the presence of xylene furnished enaminone **3**. Reaction of enaminone **3** with *N*-nucleophiles, such as urea derivatives in dioxane or EtOH/DMF mixture under reflux for four to six hours in the presence of a catalytic amount of ZnCl_2_ as a Lewis acid afforded **4a**–**c**. The formation of compounds **4a**–**c** would involve an initial addition of the amino group in urea to the activated double bond in enaminone derivative **3**, followed by deamination to an intermediate, which then undergoes cyclization and aromatization via loss of water, affording the final isolable product. Similarly, enaminone derivative **3** cyclized with hydrazine compounds by refluxing with absolute ethanol for six hours. The novel bispyrazole **5a** was assumed to be formed via addition of the amino group in the hydrazine to the activated double bond of the enamine derivative, followed by deamination, dehydration, and subsequently nucleophilic cyclization to afford the final product. The utility of enaminone **3** in the synthesis of annelated heterocycles was further explored via its reaction with 4-amino-1,2,4-triazole in absolute ethanol under reflux for seven hours in the presence of a catalytic amount of ZnCl_2_ affording compound **6**. The study was extended to investigate the behavior of enaminone derivatives **3** with different nucleophiles such as 2-aminobenzimidazole in order to synthesize various heterocyclic ring systems. Thus, the reaction of **3** with 2-iminobenzimidazole in refluxing ethanol in the presence of catalytic amount of ZnCl_2_ furnished the corresponding product **7**. The structures of these compounds were determined by ^1^H-NMR, EI, IR and UV spectroscopic, and micro analyses for carbon, hydrogen and nitrogen [22].

### 2.2. Biological Activity Evaluation

Compounds **3**, **4b**, **5a**, **5b** and **7** were evaluated for a variety of biological activities including β-glucuronidase and α-glucosidase inhibiton, antioxidant, and anticancer properties by using non-physiologica and *in vitro* biochemical and mechanism-based assays. The results of the assays are presented in Table 1.

Compound **4b** (IC_50_ = 1.3 ± 0.172 μM) was found to be most potent inhibitor of β-glucuronidase enzyme, more active than the standard, d-saccharic acid 1,4-lactone (IC_50_ = 45.8 ± 2.5 μM). This compound was found to be inactive in DPPH radical scavenging and α-glucosidase inhibition assays. However interesting results were obtained for pyrazole ring containing compounds **5a** and **5b**. Both compounds showed a potent inhibition of β-glucuronidase (**5a**, IC_50_ = 2.3 ± 0.4 and **5b** IC_50_ = 8.7 ± 0.1 μM) and α-glucosidase enzymes (**5a**, IC_50_ = 22 ± 0.3 and **5b** IC_50_ = 58.4 ± 1.2 μM) again more active than standard d-saccharic acid 1,4-lactone (IC_50_ = 45.8 ± 2.2 μM) and acarbose (IC_50_ = 841 ± 1.7 μM), respectively. Compound **5b** also showed a significant antioxidant potential (IC_50_ = 165 ± 2.8 μM) in DPPH radical scavenging assay. However, phenyl substituted pyrazole ring containing compound **5a** showed no antioxidant activity. These compounds were also evaluated for their anticancer activity against PC-3 cancer cell line. Again, compound **5a** (IC_50_ = 22.5 ± 0.1 μM) showed significant antitumor activity, while all other compounds were largely inactive.

Compounds **3**–**7** were also evaluated for their inhibitory effects against other enzymes such as carbonic anhydrase, α-chymotrypsin, xanthine oxidase, and phosphodiesterase. However, no activity was observed. The results indicate that compounds **5a** and **5b** have specific and signifcant biological potential and deserve to be further investigated.

## 3. Experimental Section

All melting points were measured on a Gallenkamp melting point apparatus in open glass capillaries and are uncorrected. IR Spectra were measured as KBr pellets on a Perkin Elmer FT 1000 spectrophotometer. The NMR spectra were recorded on a Varian Mercury Jeol-400 NMR spectrometer (400 MHz) and ^13^C-NMR (100 MHz) spectra were measured in deuterated dimethylsulphoxide (DMSO-*d*_6_). Chemical shifts (δ) are referred in terms of ppm and *J*-coupling constants are given in Hz. Abbreviations for multiplicities are as follows: s (singlet), d (doublet), t (triplet), q (quartet) and m (multiplet). Mass spectra were recorded on a Shimadzu GCMS-QP 1000 EX mass spectrometer at 70 eV. Elemental analysis was carried out on an Elementar Vario EL analyzer.

### 3.1. 1,1′-(3-Methyl-4-Phenylthieno[2,3-*b*]Thiophene-2,5-Diyl)Diethanone (**2**)

A mixture of benzoylacetone **1** (16.2 g, 0.1 mol) and anhydrous potassium carbonate (25 g) in DMF (30–40 mL) was stirred vigorously at room temperature for 5 min then carbon disulfide (7.6 mL, 0.1 mol) was added with continued stirring for 30 min. The resulting mixture was cooled in ice bath, then chloroacetone (18.5 mL, 0.2 mol) was added with continued stirring for 15 min, then cooling bath subsequently removed, and the mixture was stirred for further 30 min. The solid product was collected by filtration and washed with water, dried, and the crude product was recrystallized from glacial acetic acid to obtain pale green crystals. Yield: 27.35 gm, 87 mmol, 87%; m.p. 204–206 °C; IR (ν_max_): 1645 (C=O) cm^−1; 1^H-NMR δ (ppm): 1.84 (s, 6H, CH_3_), 1.96 (s, 3H, CH_3_), 7.551–7.61 (m, 5H, C_6_H_5_); ^13^C-NMR δ (ppm): 14.49 (CH_3_), 29.37–30.55 (COCH_3_), 192.2 (C=O), 129.23, 129.55, 129.87, 134.79, 138.82, 141.84, 147.68 (Ar–C); MS *m*/*z* (%): 314 [M+, 70%], 299 (100), 226 (37), 184 (14); Anal. calcd. for C_17_H_14_O_2_S_2_: C, 64.94; H, 4.49; S, 20.40; Found: C, 64.95; H, 4.44; S, 20.43.

### 3.2. 1,1′-(3-Methyl-4-Phenylthieno[2,3-*b*]Thiophene-2,5-Diyl)Bis(3-(Dimethylamino)Prop-2-En-1-One) (**3**)

A mixture of compound **2** (1.75 g, 5 mmol), DMF-DMA (1.19 mL, 0.01 mol) was refluxed in *m*-xylene (15 mL) for 10 h. After cooling, the resulting solid product was collected by filtration to obtain dark yellow crystals. Yield: 1.55 g, 3.75 mmol, 73%; m.p. 250 °C; IR (ν_max_): 1622 (C=O) cm^−1; 1^H-NMR δ (ppm): 1.96 (s, 3H, CH_3_), 2.99 (s, 12H, CH_3_), 4.53 (d, 1H, *J* = 12.0 Hz, CH), 5.38 (d, 1H, *J* = 12.0 Hz, CH), 7.41–7.65 (m, 5H, C_6_H_5_); ^13^C-NMR δ (ppm): 14.9 (−CH_3_), 44.79 (−N=(CH_3_)_2_), 109.8 (−CO–CH=), 153.9 (=CH–N), 180 (C=O); MS *m*/*z* (%): 424 [M+, 57%], 380(51), 336 (18), 309 (18), 98 (100); Anal. calcd. for C_23_H_24_N_2_O_2_S_2_: C, 65.06; H, 5.70; N, 6.60; S, 15.10; Found: C, 65.10; H, 5.68; S, 15.07.

### 3.3. General Procedure for the Synthesis of Compounds 4a–**c**

A mixture of compound **3** (0.212 g, 0.5 mmol), urea dervitives (2 equiv., 1 mmol) refluxed in dioxane (20 mL) for 4–6 h after in the presence of 0.5 mL of TEA and catalytic amount of ZnCl_2_. After cooling, the resulting solid products were filtered off, washed with ethanol, dried, and recrystallized from DMF/EtOH, afford the corresponding derivatives **4a**–**c**.

#### 3.3.1. 4,4′-(3-Methyl-4-Phenylthieno[2,3-*b*]Thiophene-2,5-Diyl)Dipyrimidin-2-Ol (4a)

Compound **4a** was prepared from urea, following general procedure Section 3.3, as a pale yellow crystals powder. Yield: 0.14 g, 0.33 mmol, 67%; m.p. 248 °C; IR (ν_max_): 3444 (OH), 1624 (C=N) cm^−1; 1^H-NMR δ (ppm): 1.96 (s, 3H, CH_3_), 5.38 (d, 1H, *J* = 7.8 Hz, CH), 6.5 (s, 1H, O–H), 7.65 (d, 1H, *J* = 7.8 Hz, CH), 7.41–7.65 (m, 5H, C_6_H_5_); MS *m*/*z* (%): 418 [M+, 2%]; Anal. calcd. for C_21_H_14_N_4_O_2_S_2_: C, 60.27; H, 3.37; N, 13.39; O, 7.65; S, 15.32; Found: C, 60.24; H, 3.31; N, 13.38; S, 15.32.

#### 3.3.2. 4,4′-(3-Methyl-4-Phenylthieno[2,3-*b*]Thiophene-2,5-Diyl)Dipyrimidin-2-Thiol (4b)

Compound **4b** was prepared from thiourea following general procedure Section 3.3, as a pale yellow crystals powder. Yield: 0.15 g, 0.34 mmol, 68%; m.p. 247 °C; IR (ν_max_): 1625 (C=N) cm^−1; 1^H-NMR δ (ppm): 1.96 (s, 3H, CH_3_), 5.36 (d, 1H, *J* = 7.8 Hz, CH), 6.5 (s, 1H, S–H), 7.41–7.65 (m, 5H, C_6_H_5_), 7.62 (d, 1H, *J* = 7.8 Hz, CH); MS *m*/*z* (%): 450 [M+, 2%]; Anal. calcd. for C_21_H_14_N_4_S_4_: C, 55.97; H, 3.13; N, 12.43; S, 28.46; Found: C, 55.98; H, 3.12; N, 12.41; S, 28.41.

#### 3.3.3. 4,4′-(3-Methyl-4-Phenylthieno[2,3-*b*]Thiophene-2,5-Diyl)Dipyrimidin-2-Amine (**4c**)

Compound **4c** was prepared from guanidine following general procedure Section 3.3, as a yellow crystals powder. Yield: 0.15 g, 0.36 mmol, 72%; m.p. 246 °C; IR (ν_max_): 3419 (NH_2_), 1624 (C=N) cm^−1; 1^H-NMR δ (ppm): 1.96 (s, 3H, CH_3_), 4.50–4.53 (d, 2H, NH_2_), 5.39 (d, 1H, *J* = 11.7 Hz, CH), 7.41–7.52 (m, 5H, C_6_H_5_), 7.66 (d, 1H, *J* = 11.7 Hz, CH); ^13^C-NMR δ (ppm): 14.99, 19.12, 56.58, 94.12, 108, 128, 129, 130, 136, 154, 179; MS *m*/*z* (%): 416 [M+, 2%], 336 (100), 324 (47), 153 (8); Anal. calcd. for C_21_H_16_N_6_S_2_: C, 60.55; H, 3.87; N, 20.18; S, 15.40; Found: C, 60.58; H, 3.85; N, 20.15; S, 15.38.

### 3.4. General Procedure for the Synthesis of Compounds 5**a**,**b**

A mixture of compound **3** (1 mmol) and an excess of hydrazine derivatives (1 mL) were refluxed in EtOH (20 mL) for 6 h. After cooling, the resulting solid products were filtered off, washed with ethanol, dried, and recrystallized from MeOH, to obtain corresponding derivatives **5a**,**b**.

#### 3.4.1. 3,3′-(3-Methyl-4-Phenylthieno[2,3-*b*]Thiophene-2,5-Diyl)Bis(1*H*-Pyrazole) (**5a**)

Compound **5a** was prepared from hydrazine hydrate, following general procedure Section 3.4, as white crystals. Yield: 0.22 g, 0.62 mmol, 62%; m.p. 177 °C; IR (ν_max_): 3402 (NH), 1624 (C=N) cm^−1; 1^H-NMR δ (ppm): 1.87 (s, 3H, CH_3_), 6.45 (d, 1H, *J* = 4.5 Hz, CH), 7.53–7.40 (m, 5H, C_6_H_5_), 7.81 (d, 1H, *J* = 4.5 Hz, CH), 13.01 (s, 1H, NH); ^13^C-NMR δ (ppm): 14.03 (CH_3_), 103 (CH), 145 (N=CH), 128.62, 129.13, 129.96, 130.35, 130.54, 136.37, 147.33 (Ar–C); MS *m*/*z* (%): 362 [M+, 43%]; Anal. calcd. for C_19_H_14_N_4_S_2_: C, 62.96; H, 3.89; N, 15.46; S, 17.69; Found: C, 62.98; H, 3.86; N, 15.45; S, 15.72.

#### 3.4.2. 3,3′-(3-Methyl-4-Phenylthieno[2,3-*b*]Thiophene-2,5-Diyl)Bis(1-Phenyl-1*H*-Pyrazole) (**5b**)

Compound **5b** was prepared from phenyl hydrazine, following general procedure Section 3.4, as brown crystals. Yield: 0.33 g, 0.64 mmol, 64%; m.p. 199 °C; IR (ν_max_): 1606 (C=N) cm^−1; 1^H-NMR δ (ppm): 1.86 (s, 3H, CH_3_), 6.48 (d, 1H, *J* = 4.5 Hz, CH), 6.53–7.20 (m, 15H, C_6_H_5_), 7.55 (d, 1H, *J* = 4.5 Hz, CH); ^13^C-NMR δ (ppm): 14.03 (CH_3_), 102 (CH), 143 (N=CH), 128.52, 129.23, 129.94, 130.38, 130.54, 136.38, 147.33, 152.84 (Ar–C); MS *m*/*z* (%): 514 [M+, 1%]; 169 (5), 107 (100), 92 (55), 90 (35); Anal. calcd. for C_3_1H_22_N_4_S_2_: C, 72.34; H, 4.31; N, 10.89; S, 12.46; Found: C, 72.36; H, 4.29; N, 10.86; S, 12.43.

### 3.5. General Procedure for the Synthesis of Compounds **6** and **7**

To a solution of compound **3** (212 mg, 0.5 mmol) in DMF (2 mL), substituted amine (2 equiv., 1 mmol) in EtOH (20 mL, 99.9%) was added, and then the resulting reaction mixture was heated under reflux for 7 h in the presence of a catalytic amount of ZnCl_2_. After cooling, the solid product was collected by filtration, washed with ethanol, dried, and recrystallized from DMF/EtOH to afford the corresponding derivatives **6** and **7**.

#### 3.5.1. 7,7′-(3-Methyl-4-Phenylthieno[2,3-*b*]Thiophene-2,5-Diyl)Di-[1,2,4]Triazolo[1,5-*a*]Pyrimidine (**6**)

According to GP3, **6** was obtained from 4-amino-1,2,4-triazole (84 mg) as yellow crystals. Yield: 156 mg, 0.34 mmol, 67%; m.p. 245 °C; IR (ν_max_): 1624 (C=N) cm^−1; 1^H-NMR δ (ppm): 1.96 (s, 3H, CH_3_), 7.41–7.56 (m, 5H, C_6_H_5_), 8.17 (d, 1H, *J* = 8.5 Hz, CH, pyrimidyl), 8.67 (s, 1H, CH, triazole), 8.99 (d, 1H, *J* = 12.5 Hz, CH, pyrimidyl); ^13^C-NMR δ (ppm): 14.29 (−CH_3_), 115.38, 125, 129.11, 129.98, 130.21, 132.81, 135.8, 152.8, 154.43, 159.98, 162.88 (Ar–C); MS *m*/*z* (%): 466 [M+, 45%]; Anal. calcd. for C_23_H_14_N_8_S_2_: C, 59.21; H, 3.02; N, 24.02; S, 13.75; Found: C, 59.19; H, 3.03; N, 24.04; S, 13.71.

#### 3.5.2. 2,2′-(3-Methyl-4-Phenylthieno[2,3-*b*]Thiophene-2,5-Diyl)Bis(Benzo[4,5]Imidazo[1,2-*a*] Pyrimidine) (**7**)

According to general procedure Section 3.1.5, **7** was obtained from 2-aminobenzimidazole (133 mg) as dark yellow crystals. Yield: 175 mg, 0.31 mmol, 62%; m.p. 245 °C; IR (ν_max_): 1622 (C=N) cm^−1; 1^H-NMR δ (ppm): 1.96 (s, 3H, CH_3_), 7.42–7.65 (m, 8H, C_6_H_5_, benzimidazole), 8.16 (d, 1H, *J* = 12.5 Hz, CH, pyrimidyl), 8.39 (d, 1H, *J* = 8.5 Hz, CH, benzimidazole), 8.80 (d, 1H, *J* = 12.5 Hz, CH, pyrimidyl); ^13^C-NMR δ (ppm): 14.8 (−CH_3_), 112.0, 115.1, 122.5, 127.1, 128.1, 129.9, 131.31, 135.97, 139.1, 142.0, 148.4, 156 (Ar–C); MS *m*/*z* (%): 564 [M+, 1%], 488 (1.5), 380 (38), 336 (55), 324(67), 98 (100); Anal. calcd. for C_33_H_20_N_6_S_2_: C, 70.19; H, 3.57; N, 14.88; S, 11.36; Found: C, 70.21; H, 3.54; N, 14.85; S, 11.40.

### 3.6. Biological Activities

Results are presented here as means ± standard error mean from triplicate (*n* = 3) observation. IC_50_ Values were determined by using EZ-FIT, enzyme kinetics software by Perrella Scientific, Inc., USA.

#### 3.6.1. Anticancer Activity

Cytotoxic activity of compounds was evaluated in 96-well flat-bottomed microplates by using the standard MTT (3-[4,5-dimethylthiazole-2-yl]-2,5-diphenyl-tetrazolium bromide, MP) colorimetric assay [22]. For this purpose, PC3 cells (Prostrate Cancer) were cultured in Dulbecco’s Modified Eagle Medium, supplemented with 10% of fetal bovine serum (FBS, PAA), 100 IU/mL of penicillin and 100 μg/mL of streptomycin in 75 cm^2^ flasks, and kept in 5% CO_2_ incubator at 37 °C. Exponentially growing cells were harvested, counted with a haemocytometer, and diluted with a particular medium with 5% FBS. Cell culture with the concentration of 1 × 10^5^ cells/mL was prepared and introduced (100 μL/well) into 96-well plates. After overnight incubation, medium was removed and 200 μL of fresh medium was added with different concentrations of compounds (1–30 μM). Stock solution, 20 mM of compounds were prepared in 100% DMSO, and the final concentration of DMSO at 30 μM is 0.15%. After 48 h, 200 μL MTT (0.5 mg/mL) was added to each well and incubated further for four hours. Subsequently, 100 μL of DMSO was added to each well. The extent of MTT reduction to formazan within cells was calculated by measuring the absorbance at 570 nm, using a microplate reader (SpectraMax plus, Molecular Devices, Sunnyvale, CA, USA). The cytotoxicity was recorded as concentration causing 50% growth inhibition (IC_50_) for PC3 cells. The percent inhibition was calculated by using the following formula:

$$\begin{array}{l} \%\;\text{Inhibition}=100-((\text{mean of OD of test compound}-\text{mean of OD of negative control})/\\ (\text{mean of OD of}\;\text{positive control}-\text{mean of OD of negative}\;\text{control})\times 100). \end{array}$$

The results (% inhibition) were processed by using SoftMax Pro software (Molecular Device, USA).

#### 3.6.2. *In Vitro* Antioxidant Activity

Test samples were allowed to react with stable free radical, 1,1-diphenyl-2-picrylhydrazyl radical (DPPH, Wako Chemicals, Shanghai, China) for half an hour at 37 °C. Various concentrations of test samples (prepared in DMSO) were incubated with DPPH (300 μM; prepared in ethanol). After incubation, decrease in absorption was measured at 515 nm by using a microplate reader (SpectraMax plus 384). Percentage radical scavenging activity (% RSA) of samples was determined in comparison with a DMSO-treated control group. Percentage of radical scavenging activity was calculated by using the formula given in statistical analysis section [23].

#### 3.6.3. *In Vitro* β-Glucuronidase Inhibition Assay

β-Glucuronidase inhibitory activity was determined by the spectrophotometric method by measuring the absorbance at 405 nm of *p*-nitrophenol formed from the substrate *p*-nitrophenyl-β-d-glucuronide N1627-250 mg (Sigma Aldrich Co., 3050 spruce street, St. Louis, MO, USA). The total reaction volume was 250 μL. The compound (5 μL) was dissolved in DMSO (100%), which became 2% in the ultimate assay (250 μL) and similar conditions were used for standard (d-saccharic acid 1,4-lactone, Sigma Aldrich Co.). The reaction mixture contained 185 μL of 0.1 M acetate buffer, 5 μL of test compound solution, 10 μL of (1U) enzyme solution (G7396-25KU, Sigma Aldrich) was incubated at 37 °C for 30 min. The plates were read on a multiplate reader (SpectraMax plus 384) at 405 nm after the addition of 50 μL of 0.4 mM *p*-nitrophenyl-β-d-glucuronide. All assays were performed in triplicate. IC_50_ Values were calculated by using EZ-Fit software (Perrella Scientific Inc., Amherst, MA, USA). These values are the mean of three independent readings [24].

#### 3.6.4. *In Vitro* α-Glucosidase Inhibition Assay

α-Glucosidase inhibition assay was performed spectrophotometrically. α-Glucosidase from *Saccharomyces cerevisiae* (G0660-750UN, Sigma Aldrich), was dissolved in phosphate buffer (pH 6.8, 50 mM). Test compounds were dissolved in 70% DMSO. In 96-well plates, 20 μL of test sample, 20 μL of enzyme and 135 μL of buffer were added and incubated for 15 min at 37 °C. After incubation, 25 μL of *p*-nitrophenyl-α-d-glucopyranoside (0.7 mM, Sigma Aldrich) was added and change in absorbance was monitored for 30 min at 400 nm. Test compound was replaced with DMSO (7.5% final) in control. Acarbose (Sigma Aldrich) was used as a standard inhibitor [25,26].

#### 3.6.5. Statistical Analysis

All reactions were performed in triplicate in a final volume of 200 μL. The results were processed using SoftMax Pro 4.8 software (Molecular Devices, Sunnyvale, CA, USA) and then using MS Excel. The percentage of inhibition was calculated by the following formula:

% Inhibition=100-(OD of test sample/OD of the control)×100

## 4. Conclusions

In summary, the present investigation describes an efficient method for the synthesis of novel *bis*heterocycles, many of which may display interesting biological activities in the field of medicinal chemistry. Compounds **4b**, **5a**,**b** showed the inhibitory effect against β-glucuronidase enzyme, which is highly expressed in cancer, rheumatoid arthritis, and AIDS. Compounds **5a** and **5b** also showed a potent inhibition of α-glucosidase enzyme and can serve as potential lead molecules for further investigation.

## Figures and Tables

**Table 1:** Results of various biological assays on compounds **3**–**7**.

Compounds	IC_50_ ± SEM [μM]			

	Anticancer Activity (PC-3 Cell line)	DPPH Radical Scavenging Assay	β-Glucuronidase Inhibition	α-Glucosidase Inhibition
**3**	>30	NA	NA	NA
**4a**	>30	**-**	**-**	**-**
**4b**	>30	NA	**1.3 ± 0.2**	**-**
**4c**	**-**	**-**	**-**	**-**
**5a**	**22.5 ± 0.1**	NA	**2.3 ± 0.4**	**22.0 ± 0.3**
**5b**	>30	**165 ± 2.8**	**8.7 ± 0.1**	**58.4 ± 1.2**
**6**	>30	**-**	**-**	**-**
**7**	>30	-	NA	NA
**Std.**	**Doxorubicin**	***N*-Acetylcysteine**	**d-Saccharic acid**	**Acarbose**
	**0.91 ± 0.1**	**106 ± 1.1**	**1,4-lactone 45.8 ± 2.2**	**841 ± 1.7**

SEM = standard error of mean; NA = not active; failed to show 50% or more inhibition at 500 μM or more.
